# Improvement of patient-reported outcomes in severe allergic asthma by omalizumab treatment: the real life observational PROXIMA study

**DOI:** 10.1186/s40413-018-0214-3

**Published:** 2018-11-01

**Authors:** Giorgio Walter Canonica, Paola Rottoli, Caterina Bucca, Maria Cristina Zappa, Giovanni Michetti, Bruno Macciocchi, Cristiano Caruso, Pierachille Santus, Marta Bartezaghi, Laura Rigoni, on behalf of PROXIMA study centers

**Affiliations:** 1 Department of Biomedical Sciences, Personalised Medicine Clinic Asthma & Allergy, Humanitas University, IRCCS Humanitas Research Hospital, Rozzano, Milan, Italy; 2 Respiratory Diseases and Lung Transplantation Unit, Department of Medical, Surgical and Neuro Sciences, University of Siena, Siena, Italy; 3 Pneumology Unit, AOU Molinette, Department of Medical Sciences, University of Turin, Turin, Italy; 4 Division of Lung Disease, Sandro Pertini Hospital, Rome, Italy; 5 Pneumology Unit, Habilita San Marco, Bergamo, Italy; 6 Pneumology Unit, Hospital St. Scholastica, Cassino, Italy; 7 Allergy Unit, Fondazione Policlinico Gemelli, Presidio Columbus, Rome, Italy; 8 Division of Respiratory Diseases, “L.Sacco” University Hospital, ASST Fatebenefratelli-Sacco, Department of Biological and Clinical Sciences (DIBIC), University of Milan, Milan, Italy; 9 Novartis Farma SpA, Largo U. Boccioni 1, 21040 Origgio, VA Italy

**Keywords:** Omalizumab, Severe allergic asthma, Asthma control, Exacerbations, Quality of life, Perennial allergens, Seasonal allergens, Illness perception

## Abstract

**Background:**

Data on the prevalence of perennial versus seasonal allergic asthma in Italy are lacking; moreover, there is limited evidence on the effect of omalizumab on patient-reported outcomes in Italian patients with severe allergic asthma. PROXIMA, an observational, multicenter study, was designed to assess the prevalence of perennial versus seasonal allergic asthma (cross-sectional phase) and the effect of omalizumab on improving illness perception, quality of life (QoL) and asthma control of Italian patients with severe allergic asthma (longitudinal phase).

**Methods:**

The study included a cross-sectional phase (*n* = 357) and a longitudinal phase (*n* = 123): during the longitudinal phase, patients received omalizumab (75–600 mg subcutaneously every month) and were followed-up for 12 months. The primary parameter of cross-sectional phase was prevalence of perennial allergic asthma and that of longitudinal phase was proportion of patients with asthma control (assessed using asthma control questionnaire [ACQ]). Secondary parameters assessed were patients’ disease perception, level of asthma control, exacerbation rate during both cross-sectional and longitudinal phases, and patients' compliance to and persistence with omalizumab, and patients' QoL during the longitudinal phase.

**Results:**

Most patients (95.8%) had perennial allergies; 81% had polysensitization. Of 99 patients in the per-protocol set, 95 (95.96% [95% CI: 89.98–98.89%]) achieved asthma control (ACQ < 4) at both 6 and 12 months of omalizumab treatment; ACQ score decreased after 6 and 12 months (*P* < 0.0001). Omalizumab treatment resulted in a significant improvement in QoL and patients’ illness perception and 87% decrease in exacerbation rate. The compliance rate with omalizumab was high (73.2%). No new safety signals were identified during treatment.

**Conclusion:**

This study demonstrated that in severe allergic asthma, omalizumab improves patient-reported outcomes such as patients’ illness perception and QoL, while confirming improvement of asthma control and exacerbation rate reduction in Italian patients.

## Background

Asthma affects around 334 million people globally and results in ~ 250,000 deaths every year [1]. In Italy a trend toward increasing prevalence of asthma was observed between 1991 and 2010 with a 38% increase in the incidence rate [2]. According to Global Initiative for Asthma (GINA) guidelines, severe asthma is a condition requiring GINA step 4 or 5 treatment (e.g. high-dose inhaled corticosteroid/long-acting beta-2 agonist [ICS/LABA]), to prevent it from becoming uncontrolled, or asthma that remains uncontrolled despite this treatment [3]. For patients who remain uncontrolled or poorly controlled despite treatment with ICS/LABA, step-up with add-on anti-IgE has been recommended [3]. Severe asthma affects ~ 5%–10% of all patients with asthma and is characterized by poor asthma control, reduced lung function, impaired quality of life (QoL), and high risk of exacerbations and mortality [4, 5]. Almost 59%–80% of patients with severe asthma have an allergic component to their disease; allergic asthma is typically characterized by immunoglobulin E (IgE)-mediated hypersensitivity [6–11]. Sensitization to perennial allergens such as house-dust mite, pet allergens and fungus, and seasonal allergens such as pollen imposes a high risk of developing asthma [12]. Exposure to aeroallergens triggers Th2 cell-mediated immune response resulting in increased levels of IgE antibodies [13–15], which then bind to the high-affinity IgE receptors (FcεRI) on the effector cells such as mast cells and basophils. Subsequently, this results in the release of various chemical mediators leading to asthma-related symptoms such as wheezing, coughing, chest tightness and shortness of breath [16].

Omalizumab, a humanized recombinant monoclonal anti-IgE antibody, is approved in Europe, as an add-on therapy (75–600 mg subcutaneously every 2 or 4 weeks) for management of severe persistent allergic asthma in patients aged ≥6 years with symptoms inadequately controlled with high-dose ICS/LABA [17]. Omalizumab improved asthma control and QoL, with 50% reduction in severe exacerbation rate in patients with severe allergic asthma (SAA) [18]. Effectiveness of omalizumab in treating patients with SAA and improving patient-reported outcomes (PROs) has also been consistently evidenced in real-life observational studies from France, Germany, Belgium, United Kingdom, South-Eastern Mediterranean centers and Spain [19–24].

Assessment of PROs plays an important role in monitoring asthma control and in optimizing the interaction between the patients and the physicians [25, 26]. Unfortunately, patients’ viewpoint on their disease condition and treatment efficacy so far has been rather neglected. However, in the recent days, asthma management strategy moved towards a patient-centric approach reflecting more on patients’ viewpoint on their disease condition and treatment approach [27].

To date, no studies have been published on the prevalence of SAA in the Italian population; moreover, limited information is available on the effectiveness of omalizumab in treatment of SAA in Italian real-life settings. The ***PROXIMA*** (***P***atient ***R***eported ***O***utcomes and ***X***olair® ***I***n the ***M***anagement of ***A***sthma) study was designed primarily to assess the prevalence of perennial allergic asthma in the Italian population during the cross-sectional phase and to determine the proportion of patients with SAA treated with omalizumab who achieved and sustained asthma control over 12 months in the longitudinal phase [28]. The secondary objectives included assessment of patients' disease perception, level of asthma control, and rate of exacerbations during both cross-sectional and longitudinal phases of the study, and patients' compliance to and persistence with omalizumab, and patients' QoL during the longitudinal phase.

## Methods

### Study design and patients

PROXIMA was an observational, two-phase study conducted from 27th December, 2013 to 21st June, 2016 at 25 outpatient settings in Italy (hospitals and university centers specialized in asthma treatment) [28]. The study consisted of a cross-sectional phase and a prospective longitudinal phase. Patients aged ≥18 years, diagnosed with SAA, who were at step 4 as per GINA guidelines and required a therapeutic step-up, were included in the cross-sectional phase. Patients who started treatment with omalizumab as per clinician judgement (according to AIFA criteria) at baseline visit were included in the longitudinal phase. Patients started omalizumab treatment not earlier than 15 days before enrolment, and within 90 days after enrolment and were followed-up for 12 months.

Patients were excluded if they were unable to complete the patient questionnaire or were involved in any experimental study during the study entry.

Patients received omalizumab as per clinical practice [17] and were followed-up for 12 months. The follow-up visits were scheduled at 6 and 12 months.

The study was conducted in accordance with the ethical principles laid down in the Declaration of Helsinki and the Italian Medicines Agency (AIFA) Guideline for classification and management of observational studies on drugs [29–31]. All patients provided informed consent before participating in the study.

### Assessments

The primary objectives were to determine: (i) proportion of patients with perennial versus seasonal allergic asthma based on the clinician’s judgment and a skin prick test or an *in vitro* test (at baseline, cross-sectional phase) and (ii) proportion of patients who achieved and maintained asthma control (at 6 and 12 months) with omalizumab (longitudinal phase). Asthma control was evaluated using Asthma Control Questionnaire (ACQ), a validated tool with a 7-point scale (score 0: well-controlled, score 6: extremely poorly controlled). Patients with an ACQ score < 4 at both 6 and 12 months after treatment were considered as “responders”. Patients with an ACQ score < 4 at either 6 or 12 months were classified as “controlled” patients, and those with an ACQ score < 1 at either 6 or 12 months were classified as “fully controlled” [32].

Secondary objectives for cross-sectional phase included: level of asthma control, patients’ disease perception (using Brief Illness Perception Questionnaire [BIPQ, a 9-item questionnaire]), and exacerbation rate at baseline in the overall population and in patients with perennial versus seasonal asthma. Secondary parameters for longitudinal phase included: proportion of patients with ≥1 episode of asthma exacerbation during 12-month treatment period; patients’ disease perception and QoL (using EuroQoL five-dimensional three-level questionnaire [EQ-5D-3 L]) at 6 and 12 months; patients’ compliance to omalizumab at 6 and 12 months; and, patients’ persistence with omalizumab treatment during 12 months. Safety assessments included recording of adverse events (AEs).

### Statistical analysis

PROXIMA was an observational study with no confirmatory aims. Overall, 357 patients and 99 patients, respectively, were evaluable for the primary objective of the cross-sectional and longitudinal phase. Considering the sample size of 357 patients for the cross-sectional phase, the maximum allowable precision of the estimate (i.e. width of 95 confidence interval [CI]) reached when the proportion is 50%, is 10.4%. With respect to the longitudinal phase, the maximum allowable precision of the estimate, considering 99 evaluable patients, is 19.6%; this precision increased to 11.8%, when the proportion is equal to 90% (i.e. that observed in the study). In accordance with the aforementioned statement, the precision of the estimate was < 15%.

The data on primary parameters were descriptively summarized, and corresponding 95% CIs were presented. Wilcoxon nonparametric test was performed to compare BIPQ domain scores and ACQ total scores between patients with perennial and seasonal asthma. Fisher’s exact test was used for frequency of patients with good–moderate control (ACQ < 4) or with poor–very poor control (ACQ ≥4) [32] in patients with perennial versus seasonal asthma. Paired sample t-test (or nonparametric signed rank test) was performed to assess changes in ACQ scores, EuroQoL Visual Analog Scale (EQ-VAS), and BIPQ domain scores from baseline to 6 and 12 months. Kaplan–Meier survival curve analysis was performed to evaluate persistence with omalizumab treatment during the 12-month follow-up period.

The cross-sectional population included all enrolled patients who met inclusion and exclusion criteria for the cross-sectional phase; and the longitudinal population included all patients enrolled in the longitudinal phase as defined in the key inclusion criteria; and the per-protocol population included all evaluable patients who completed the longitudinal phase and for whom ACQ score at 6 and/or 12 months was computable.

### Sensitivity analysis

To evaluate omalizumab effects on asthma control, considering also patients with missing ACQ total scores at one of the follow-up visits, the concepts of “worst scenario” and “best scenario” were adopted. In worst scenario analysis, patients with missing ACQ total scores at 6- or 12-month follow-up visits were considered as “not controlled”. In best scenario analysis, patients with missing ACQ total scores were considered “controlled” if ACQ scores were < 4 in any one assessment, and patients were not dropped out owing to efficacy.

## Results

### Patient demographics and clinical characteristics

**Fig. 1:**
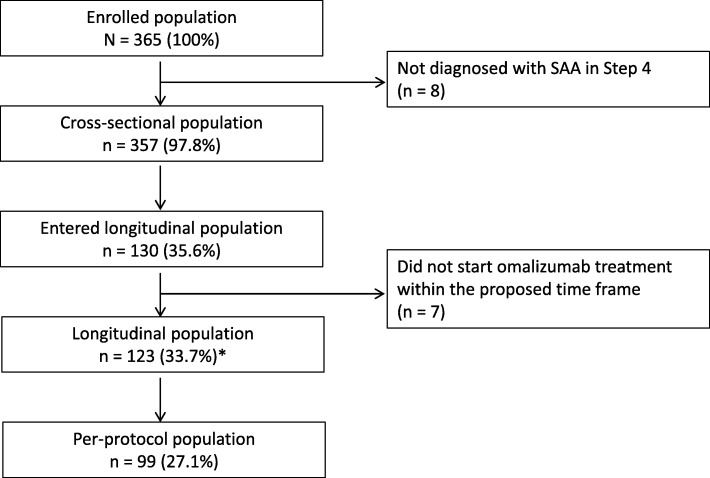
Patient disposition. *Of 123 patients, 104 completed the longitudinal phase and 19 discontinued, 8 of whom were lost to follow-up, 7 patients voluntarily discontinued the study and 4 patients withdrew for to other reasons. (SAA, severe allergic asthma)

Of 365 patients enrolled, 357 contributed to the cross-sectional population set, and 123 to the longitudinal population set (Fig. 1). The majority of patients in both phases were women (62%–65%) and Caucasians (~ 95%) with mean asthma duration of ~ 19 years, and had at least one co-morbidity (> 58%; Table 1). Cardiovascular diseases were the most frequent comorbidities in both the population sets (~ 21%), followed by chronic rhinitis in the cross-sectional population (~ 15%) and chronic sinusitis/rhinosinusitis in the longitudinal population. Mean number of asthma exacerbations during the 12 months before enrolment was 3.6 in the cross-sectional population and 4.6 in the longitudinal population (Table 1).

**Table 1:** Demographic and baseline data and clinical characteristics (cross-sectional population and longitudinal population sets)

Characteristic	Cross-sectional population *N* = 357	Longitudinal population *N* = 123
Female	232 (65.0)	76 (61.8)
Caucasian	340 (95.2)	117 (95.1)
Age, years (mean ± SD)	50.5 ± 15.5	52.7 ± 13.6
At least one comorbidity	208 (58.3)	77 (62.6)
Asthma duration, years (mean ± SD)^a^	18.4 ± 14.9	19.8 ± 14.5
Age at diagnosis, years (mean ± SD)^a^	32.1 ± 17.4	32.7 ± 15.8
Number of asthma exacerbations during the 12 months before enrolment^b^	3.6 ± 4.2	4.6 ± 4.1
ACQ total scores^c^	2.4 ± 1.2	2.9 ± 1.1
Smoking history		
Non-smoker	265 (74.2)	90 (73.2)
Former smoker	67 (18.8)	27 (22.0)
Current smoker	25 (7.0)	6 (4.9)
FEV_1_ (L)^d^	2.0 ± 0.8	1.7 ± 0.7
IgE serum level, IU/mL, (mean ± SD)^e^	434.8 ± 556.4	409.3 ± 394.1
At least one concomitant pharmacological treatment for respiratory disease	–	122 (99.2)
Corticosteroids for systemic use	–	50 (40.7)

Data are presented as n (%), unless otherwise specified

^a^*n* = 347 in cross-sectional population and n = 119 in longitudinal population

^b^*n* = 330 in cross-sectional population and *n* = 119 in longitudinal population

^c^*n* = 339 in cross-sectional population and *n* = 96 in longitudinal population

^d^*n* = 342 in cross-sectional population and *n* = 121 in longitudinal population (assessed at baseline or within 3 months before enrolment)

^e^*n* = 252 in cross-sectional population and *n* = 121 in longitudinal population (assessed at baseline or within 3 months before enrolment)

*ACQ* asthma control questionnaire, *FEV*_*1*_ forced expiratory volume in one second, *IgE* immunoglobulin E, *SD* standard deviation

### Cross-sectional phase

#### Prevalence of perennial versus seasonal allergic asthma

Based on clinical judgment, 95.8% (*n* = 342) patients had perennial allergies (95% CI: 93.2–97.6%), and 4.2% (*n* = 15) patients had seasonal allergies (95% CI: 2.4–6.8%). A similar trend was observed based on confirmatory allergy test: 83.8% (*n* = 299) patients had perennial allergies and 10.1% (*n* = 36) patients had seasonal allergies. The majority of patients (81% [*n* = 289]) were positive to more than one allergen (polysensitization). The most common trigger for perennial asthma, as judged by the physician, was dermatophagoides (74.3% [*n* = 248]), and for seasonal asthma was grasses (60.0% [*n* = 9]; Table 2**)**.

**Table 2:** Frequency of aeroallergens in ≥20% of patients with perennial and seasonal allergic asthma (according to clinician judgement) (cross-sectional population)

Allergen	Patients with perennial allergens (*N* = 342)	Patients with seasonal allergens (*N* = 15)
Grasses	174/315 (55.2%)	9/15 (60.0%)
Pellitory	134/319 (42.0%)	4/14 (28.6%)
Birch	67/288 (23.3%)	3/14 (21.4%)
Hazel	46/269 (17.1%)	3/14 (21.4%)
Cypress	66/285 (23.2%)	4/13 (30.8%)
Olive tree	92/311 (29.6%)	3/13 (23.1%)
Dermatophagoides	248/334 (74.3%)	2/14 (14.3%)
Cat epidermal allergen	97/305 (31.8%)	1/14 (7.1%)
Dog epidermal allergen	66/299 (22.1%)	1/14 (7.1%)

#### Patients’ perception of asthma

**Fig. 2:**
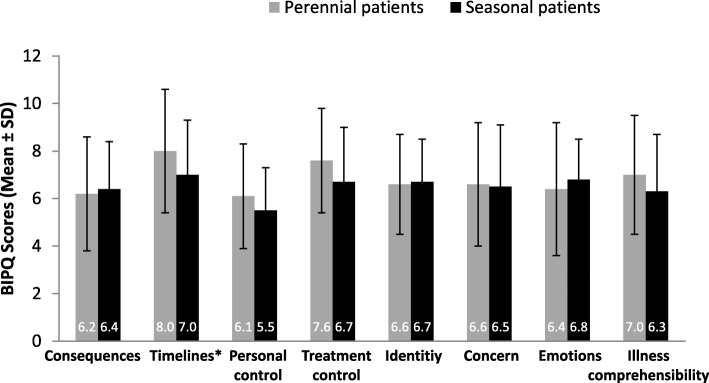
Patients’ illness perception, assessed by BIPQ at baseline (cross-sectional population). *P = 0.041 for perennial vs seasonal asthma patients; *p* values are non-significant for the remaining components. *P-*values were calculated using Wilcoxon test. BIPQ, Brief Illness Perception Questionnaire

Perception of illness was comparable between patients with perennial and seasonal asthma for all BIPQ domains**,** except the “timelines” domain (*P* = 0.0414). These data suggest that patients with perennial asthma perceive their duration of illness to be longer than those with seasonal asthma (Fig. 2), which was to be expected because perennial allergies last year-round.

#### Asthma control

The majority of patients (89.7% [*n* = 304]) demonstrated good–moderate level of asthma control (ACQ < 4), with a mean ± SD ACQ total score of 2.4 ± 1.2. No significant difference was observed in ACQ total scores in patients with perennial and seasonal allergies (mean ± SD: 2.4 ± 1.2 [*n* = 325] vs. 2.3 ± 1.3 [*n* = 14]; *P* = 0.7038).

#### Asthma exacerbations

The mean ± SD number of asthma exacerbations during 12 months before enrolment was 3.6 ± 4.2 and the proportion of patients with ≥1 asthma exacerbation in the 12 months before baseline visit was 87.6% (*n* = 289/330). Exacerbation rates were similar in patients with perennial and seasonal asthma (3.6 ± 4.2 vs. 3.0 ± 2.3; *P* = 1.000). Most patients with perennial (87.5% [*n* = 279/319]) and seasonal (90.9% [*n* = 10/11]) asthma experienced ≥1 exacerbation in the 12 months before baseline visit.

### Longitudinal phase

#### Patients’ perception of asthma

Omalizumab significantly improved most of the components of BIPQ questionnaire after 6 and 12 months (Table 3). Patients reported to have experienced better asthma control, to have a less severe impact of asthma on their lives, to have experienced less symptoms from their illness, and to be less concerned and emotionally affected by their illness. Overall, patients’ QoL improved during treatment with omalizumab.

**Table 3:** Illness perception (assessed by BIPQ) following omalizumab treatment at each time point (longitudinal population)

Component	Baseline	Change at 6 months from baseline	*P*-value	Change at 12 months from baseline	*P*-value
Consequences	7.3 ± 1.9	−2.0 ± 2.4	< 0.0001	−2.2 ± 2.7	< 0.0001
Timeline	8.1 ± 2.4	−1.0 ± 2.5	< 0.0001	−0.3 ± 2.4	0.1938
Personal control	5.9 ± 2.0	0.7 ± 2.3	0.0005	0.9 ± 2.5	0.0003
Treatment control	7.2 ± 2.3	0.9 ± 2.6	0.0001	1.1 ± 2.5	< 0.0001
Identity	7.6 ± 1.7	−2.2 ± 2.5	< 0.0001	−2.0 ± 2.6	< 0.0001
Concern	7.4 ± 2.3	−1.6 ± 3.0	< 0.0001	− 1.7 ± 3.0	< 0.0001
Emotions	7.0 ± 2.6	−1.2 ± 2.8	< 0.0001	−1.1 ± 3.0	< 0.0001
Illness comprehensibility	7.1 ± 2.3	0.4 ± 2.3	0.0690	0.7 ± 2.4	0.0076

Data presented as mean ± SD

*P* < 0.05 is considered as statistically significant. *P*-value was calculated using Wilcoxon test

*BIPQ* Brief Illness Perception Questionnaire

#### Quality of life

Compared with baseline, a greater proportion of patients reported no problem in mobility, self-care, performing usual activities, pain/discomfort, and anxiety/depression during 6- and 12-month follow-up periods, as assessed by Euro-QoL 5D-3 L (Table 4). Moreover, omalizumab significantly improved (*P* < 0.0001) the QoL, as shown by VAS score changes at 6 months (+ 13.9 ± 17.4) and at 12 months (+ 15.4 ± 20.3) compared with baseline, assessed by EQ-VAS.

**Table 4:** Patients with improved quality of life – EQ-5D-3 L questionnaire (longitudinal population)

Parameter	Baseline (*N* = 121)	6 months (*N* = 113)	12 months (*N* = 103)
No problems in walking	47.9	73.5	72.8
No problems in self-care	74.4	91.2	90.3
No problems in performing usual activities^a^	36.7	58.4	65.0
No pain/discomfort^b^	31.4	61.9	63.7
No anxiety/depression	34.7	57.5	57.3
VAS scores, mean ± SD	55.1 ± 18.4	69.2 ± 15.9	71.0 ± 16.0

Data are presented as % of patients, unless otherwise specified

^a^*N* = 120 at baseline; ^b^*N* = 102 at 12 months

*P* < 0.0001 for VAS score changes from baseline to 6 months as well as 12 months. *P*-value was calculated using paired t-test

*EQ-5D-3 L* EuroQoL five-dimensional three-level questionnaire, *SD* standard deviation, *VAS* Visual Analogue Scale

#### Proportion of patients with asthma control

**Fig. 3:**
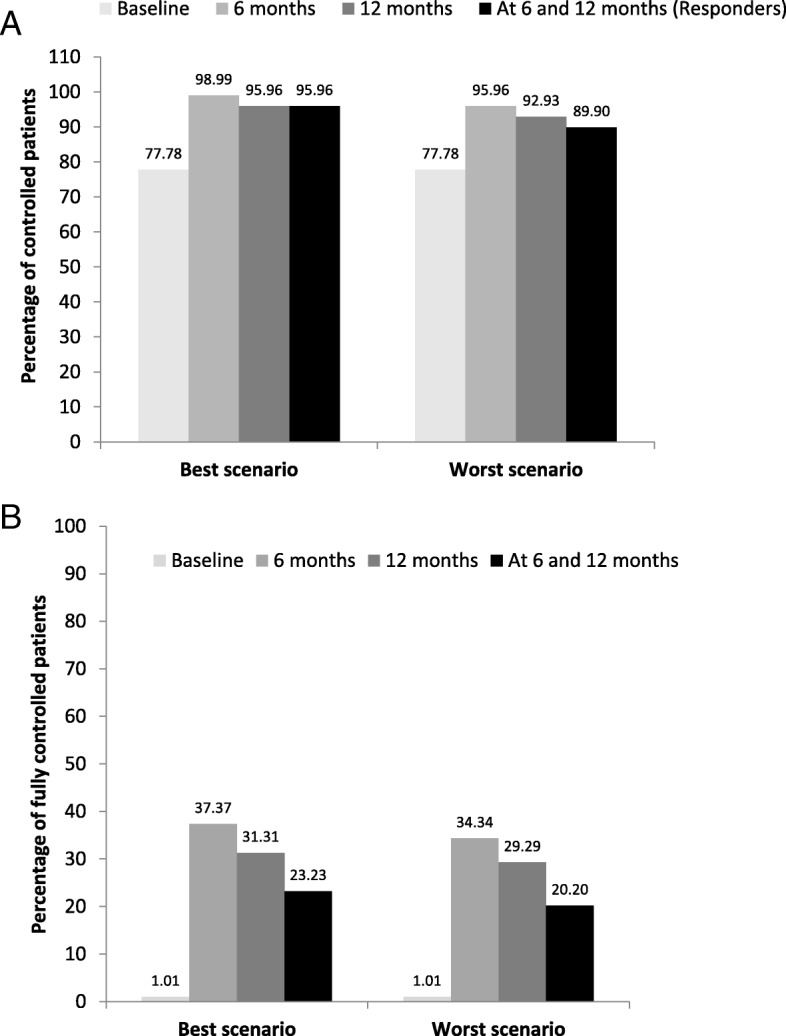
Percentage of (**a**) controlled (ACQ < 4) and (**b**) fully controlled patients (ACQ < 1) in the worst and best scenarios. Best scenario: Patients with missing ACQ total scores were considered "controlled" if ACQ scores were <4 in any one assessment, and patients were not dropped out owing to efficacy. Worst scenario: Patients with missing ACQ total scores at 6 or 12 months follow-up visits were considered "not controlled".  *Responders were patients who achieved asthma control (ACQ < 4) at both 6 and 12 months. ACQ, Asthma Control Questionnaire

Asthma control following omalizumab treatment was assessed in the per-protocol population (*n* = 99). Most patients achieved disease control at 6 months, which was maintained until 12 months (responders) after omalizumab treatment (best scenario: 95.96%, *n* = 95/99, 95% CI: 89.98–98.89%; worst scenario: 89.90%, *n* = 89/99, 95% CI: 82.21–95.05%). Of note, ~ 20–23% of patients achieved full asthma control (ACQ < 1) at both 6 and 12 months of treatment (best scenario: 23.23%, *n* = 23/99, 95% CI: 15.33–32.79%; worst scenario: 20.20%; *n* = 20/99, 95% CI: 12.80–29.46%; Fig. 3).

#### Asthma control – ACQ scores

A significant improvement (*P* < 0.0001) in mean ACQ total scores and individual domain scores from baseline was observed at 6 and 12 months (Table 5). Of particular note, an improvement in lung function, as reflected by an increase in forced expiratory volume in 1 s (FEV_1_), was observed at 6 and 12 months. At baseline, patients had a mean FEV_1_ of 1.7 L which increased by 400 mL at 6 months and by 500 mL at 12 months.

**Table 5:** Summary statistics of asthma control (assessed by ACQ) at each time point (per-protocol population)

	Baseline	6 months	12 months	Change from baseline to 6 months; *p* value	Change from baseline to 12 months; *p* value
ACQ total score	2.9 ± 1.1	1.4 ± 1.0	1.6 ± 1.1	−1.5 ± 1.2; *P* < 0.0001	−1.4 ± 1.1; *P* < 0.0001
Item 1	2.1 ± 1.6	1.0 ± 1.3	1.1 ± 1.2	−1.1 ± 1.6; *P* < 0.0001	−1.0 ± 1.7; *P* < 0.0001
Item 2	3.1 ± 1.7	1.3 ± 1.2	1.5 ± 1.4	−1.8 ± 1.8; *P* < 0.0001	−1.6 ± 1.9; *P* < 0.0001
Item 3	3.2 ± 1.4	1.3 ± 1.2	1.5 ± 1.3	−1.9 ± 1.7; *P* < 0.0001	−1.7 ± 1.7; *P* < 0.0001
Item 4	3.7 ± 1.6	1.7 ± 1.5	1.8 ± 1.5	−1.9 ± 1.9; *P* < 0.0001	−1.9 ± 1.8; *P* < 0.0001
Item 5	3.1 ± 1.8	1.4 ± 1.5	1.6 ± 1.7	−1.7 ± 1.7; *P* < 0.0001	−1.6 ± 1.9; *P* < 0.0001
Item 6	1.4 ± 1.2	0.6 ± 1.0	0.6 ± 0.9	−0.8 ± 1.4; *P* < 0.0001	−0.7 ± 1.2; *P* < 0.0001
FEV_1_ pre-bronchodilator (L)	1.7 ± 0.7	2.1 ± 0.9	2.2 ± 1.1	0.4 ± 0.6; *P* < 0.0001	0.4 ± 0.9; *P* < 0.0001
Item 7	4.2 ± 1.6	3.0 ± 2.0	3.0 ± 2.0	−1.2 ± 1.8; *P* < 0.0001	−1.2 ± 2.1; *P* < 0.0001

Data are presented as mean ± SD

ACQ total score was evaluated using Paired sample t-test; ACQ individual component scores were evaluated using Signed Rank test

*P* < 0.05 is considered statistically significant

*ACQ* Asthma Control Questionnaire

#### Asthma exacerbations

The proportion of patients experiencing ≥1 asthma exacerbation was reduced following omalizumab treatment during 12 months. Of 121 patients, 33 (27.27%; 95% CI: 19.57–36.12%) experienced ≥1 asthma exacerbation during the 12 months following treatment compared with 114 of 119 patients (95.8%) who experienced ≥1 asthma exacerbation in the 12 months before baseline visit. The mean ± SD number of exacerbation episodes per patient reported during the 12 months before enrolment was 4.6 ± 4.1 and omalizumab significantly (*P* < 0.0001) reduced the rate of exacerbations by 87% to 0.6 ± 1.2 (*n* = 121) at 12 months, with a total mean ± SD change of − 4.0 ± 4.2 (*n* = 117) in number of exacerbations from baseline.

#### Patient compliance to omalizumab and treatment persistence

A mean ± SD compliance rate of 96.9 ± 7.8% (*n* = 123) was observed for omalizumab during 12-month follow-up period. The maximum exposure time to omalizumab was 407.0 days. Of note, majority of patients remained in treatment with omalizumab during the whole observation period: 102 patients (82.9%) during the first 6-month follow-up period, 96 (88.1%) during the second 6-month follow-up period, and 90 (73.2%) considering the overall 12 months of follow-up. Only nine (7.3%) patients reported permanent discontinuation of omalizumab and four of these patients discontinued due to lack of efficacy.

### Safety

Omalizumab was well tolerated during the study and no new/unexpected AEs were reported. Eighteen (14.6%) patients reported ≥1 AE; infections and infestations (5.7%; 7 patients), followed by respiratory, thoracic and mediastinal disorders (4.1%; 5 patients) occurred more frequently. Six (4.9%) patients reported drug-related AEs and four patients had AEs leading to treatment discontinuation. Three (2.4%) patients reported serious AEs (SAEs) – serious pelvic fracture, pulmonary edema and serious asthma; one patient (0.8%) discontinued due to SAE of pulmonary edema.

## Discussion

The PROXIMA study evaluated the prevalence of allergy to perennial and seasonal aeroallergens in Italian patients with SAA. The study also assessed the level of asthma control, patients’ illness perception, asthma exacerbations and QoL in patients with SAA. Of the total enrolled population, 123 patients received add-on omalizumab therapy in the longitudinal phase.

In general, a high degree of polysensitization to multiple perennial and seasonal allergens was observed in the Italian population. Dermatophagoides was the most frequent one, accounting for perennial allergies followed by grasses and pellitory. Seasonal allergies were attributed to frequent exposure to grasses, cypress and pellitory. These findings were in line with other studies, which report allergic sensitization to a wide range of allergens in the Italian population [33–35], contributing to high prevalence of allergic rhinitis, a condition associated with increased risk of developing asthma.

The novelty of the PROXIMA study comes from the evaluation of the disease perception and the QoL of patients by the means of PROs, which have been exploited as secondary objectives. Indeed PROs have been used only in 20 clinical trials out of ~ 300 in the last 1 year and half, but none of them was done in a real-life setting – PROXIMA study assessed these outcomes in real-life scenario [27].

Moreover, the internationally recognized global evaluation of treatment effectiveness (GETE) consider as a part of the overall assessment, the response to therapy made by the patient [36]. Thus the patient’s point of view is increasingly becoming part of the therapy evaluation. From this perspective, PROXIMA is the first study to consider the patients' disease perception together with the assessment of the QoL through the EuroQoL 5D-3 L questionnaire in patients with SAA.

While PROs can provide important insights about the burden of the disease and the efficacy of the treatment, sometimes, especially in asthma, improvement of clinical parameters may not correlate well with the patient experience [37]. But, here we have shown that the treatment of SAA with omalizumab is able to improve not only the clinical parameters including ACQ, exacerbation rate and FEV_1_ but also the PROs.

BIPQ scores reflect patients’ perception on their cognitive and emotional representation of illness [38]. Patients with either perennial or seasonal asthma reported to have experienced a discrete number of symptoms from their illness and were somehow concerned about their illness. Overall, they expressed their QoL to be quite affected by their illness, confirming the big impact that SAA can have on patients. Moreover, the level of asthma control, the proportion of patients with good-moderate asthma control and the asthma exacerbation rate were similar between patients with perennial and seasonal allergic asthma. To the best of our knowledge, this is the first study to report the effect of omalizumab on patients’ illness perception using BIPQ scores.

During the longitudinal phase, treatment with omalizumab improved all the items of BIPQ scores resulting in improved patients’ perception of the disease. Patients reported better asthma control and improved symptoms, and were less concerned and emotionally affected by their illness. Patients’ QoL also improved with omalizumab treatment, as reflected in increased VAS scores and an increase in the proportion of patients with no problems in mobility or self-care or in performing usual activities, and without pain/discomfort/anxiety/depression, as assessed by the EQ-5D-3 L. An increase in EQ-VAS scores after 12 months of omalizumab treatment was also shown in a pharmaco-epidemiological study in patients with severe persistent allergic asthma [24]. A similar trend for improvement in QoL scores was noted in an observational study (APEX II study) in the UK [22].

In this study, omalizumab demonstrated an 87% decrease in exacerbation rate from baseline and a significant reduction in the proportion of patients experiencing ≥1 exacerbation over 12 months. Consistently, in a recent Italian study [39], over 50% of patients had no exacerbations following omalizumab treatment. The eXpeRience study showed a reduction in the proportion of patients with clinically significant asthma exacerbations (from 93.2 to 45.9%) with 2-year omalizumab treatment [40]. A large longitudinal study [41], and a systematic review of 24 real-life effectiveness studies of omalizumab [42], have reported remarkably diminished rates of asthma exacerbations with omalizumab treatment. During the pre-omalizumab period, a high exacerbations rate has been experienced by the patients associated with poor QoL, and after omalizumab treatment, a reduction in exacerbation rate along with an improvement in QoL was observed. Based on this, it can be hypothesized that significant reduction in exacerbations may be the major determinant of the observed improvement in patients’ QoL.

Treatment with omalizumab showed a significant improvement in asthma control at 6 and 12 months. These results were consistent with the data from a real-world global study – the eXpeRience study [40], and other similar real-life studies in Spain and Portugal [3, 20], where omalizumab treatment resulted in improved asthma control. In the PROXIMA study, the proportion of responders (ACQ < 4) was high with omalizumab treatment, with a response rate of 89–96%. These data were well supported by results from the eXpeRience and the XCLUSIVE study [40, 43], where ~ 70–79% of patients were responders (achieved excellent/good response as analyzed by GETE) after 4 months of omalizumab treatment. Importantly, an appreciably high response rate to omalizumab was obtained despite high comorbidities in this population and more severe disease, indicating omalizumab’s effectiveness in SAA patients with comorbidities. Furthermore, omalizumab also showed significant improvement in each item of the ACQ, demonstrating omalizumab benefits in improving lung function and reducing night-time awakenings, symptoms, and rescue medication use without loss of asthma control.

A high compliance rate (97%) to omalizumab was observed during the 12-month treatment period in the PROXIMA study with 90 (73.2%) patients persisting with the treatment (not withdrawing from the study either permanently or temporarily); only 9 patients reported permanent discontinuation. These beneficial effects could be attributed to reduced exacerbation rates and low incidence of AEs and SAEs observed with omalizumab treatment. In particular, no cardiovascular/cerebrovascular AEs were reported with omalizumab. This trend was similar to that in a retrospective study where ~ 65% of patients were compliant to omalizumab and 54% of omalizumab users were persistent with treatment [44]. Overall, the safety data reported in this study are concordant with the known safety profile of omalizumab and the current omalizumab summary of product characteristics (SmPC)-reported findings [17].

### Limitations

The study design used for PROXIMA study may impose a potential risk of selection bias owing to exclusion of patients who are unable to complete the patient questionnaires. However, this choice was necessary to satisfy the study objectives.

## Conclusions

In the PROXIMA study, omalizumab significantly improved illness perception and QoL after 12 months of treatment. The study revealed a wide-spread trend of allergy to perennial allergens among SAA patients in Italy. The study demonstrated the effectiveness of omalizumab in SAA patients by improving asthma control and decreasing exacerbation rate. Of note, patient compliance to omalizumab was high and the effectiveness of omalizumab was observed in a very severe group of patients.

## Abbreviations

ACQ: Asthma Control Questionnaire
AEs: Adverse events
BIPQ: Brief Illness Perception Questionnaire
CI: Confidence interval
EQ-5D-3 L: EuroQoL five-dimensional three-level questionnaire
EQ-VAS: EuroQoL Visual Analog Scale
FEV_1_: Forced expiratory volume in 1 s
GETE: Global evaluation of treatment effectiveness
GINA: Global Initiative for Asthma
ICS: Inhaled corticosteroid
IgE: Immunoglobulin E
LABA: Long-acting beta-2 agonist
PROs: Patient-reported outcomes
QoL: Quality of life
SAA: Severe allergic asthma
SAEs: Serious adverse events
SD: Standard deviation
SmPC: Summary of product characteristics
VAS: Visual Analog Scale
